# Challenges facing the veterinary profession in Ireland: 1. clinical veterinary services

**DOI:** 10.1186/s13620-017-0096-7

**Published:** 2017-06-05

**Authors:** Manuel Magalhães-Sant’Ana, Simon J. More, David B. Morton, Alison J. Hanlon

**Affiliations:** 1 Escola Universitária Vasco da Gama, Av. José R. Sousa Fernandes, Campus Universitário - Bloco B, 3020-210 Coimbra, Portugal; 2 School of Veterinary Medicine, University College Dublin, Dublin, Ireland; 3 Centre for Veterinary Epidemiology and Risk Analysis, University College Dublin, Dublin, Ireland; 4 School of BioSciences, University of Birmingham, Birmingham, B15 2TT UK

**Keywords:** 24 h care, Emergency cover, Focus group, Professional ethics, Referrals, Second opinions, Veterinary ethics, Veterinary profession

## Abstract

**Background:**

The provision of veterinary clinical services is known to elicit a range of challenges which require an ethical appraisal. In a recent Policy Delphi study, referrals/second opinions and 24 h emergency care were identified as matters of key concern by veterinary professionals in Ireland. In this case study (the first in a series of three resulting from a research workshop exploring challenges facing the veterinary profession in Ireland; the other two case studies investigate the on-farm use of veterinary antimicrobials and emergency/casualty slaughter certification) we aim to provide a value-based reflection on the constraints and possible opportunities for two prominent veterinary clinical services in Ireland: referrals/second opinions and 24 h emergency care.

**Results:**

Using a qualitative focus group approach, this study gathered evidence from relevant stakeholders, namely referral and referring veterinarians, clients, animal charities, and the regulatory body. Six overarching, interrelated constraints emerged from the thematic analysis: the need to improve current guidance, managing clients’ expectations, concerns with veterinarian well-being, financial issues, timeliness of referral, and conflicts between veterinary practices.

**Conclusions:**

Possible solutions to improve veterinary referral and out-of-hours clinical services included clarifying the terms used in current norms and regulations (namely ‘referral’, ‘second opinion’, ‘24 h emergency care’ and ‘24 h cover’), improved communication (making the client aware of the different levels of veterinary care that are being offered, and transparent and full disclosure of clinical records), and the promotion of Continuing Veterinary Education in communication, business management and ethical decision-making. These findings may help inform the Veterinary Council of Ireland about future recommendations and regulatory measures.

## Background

The provision of veterinary clinical services elicits a range of issues which require an ethical appraisal [1]. Anecdotal evidence suggest that referrals, second opinions and 24 h emergency care can pose considerable challenges to veterinary practitioners, impacting negatively on the quality of veterinary services, the protection of animal health and welfare, and the reputation of the veterinary profession [2–4].

A previous investigation, using a Policy Delphi technique, had identified referrals and second opinions, and 24 h emergency care as amongst the main ethical issues facing veterinary professionals in Ireland [5]. Under the Veterinary Council of Ireland’s Code of Professional Conduct (VCI-CPC), veterinarians “*should have no hesitation in recommending an animal owner to consult another colleague who has particular skills and/or specialised equipment, or who can provide a service not offered by the first veterinary practitioner*” (Issue 7, 11 Sept. 2014, p.28). The VCI-CPC does not attempt to define ‘referrals’ or ‘second opinions’ and the distinction between the two may not always be clear. Furthermore, guidance is lacking on how best to address the practical conflicts arising when cases are referred.

The VCI-CPC also contains provisions on 24 h emergency services, advising veterinarians to cooperate in this regard (p.19). The VCI requires “*that appropriate information be given to owners regarding the level of care that will be given to patients (*e.g., *continuous monitoring, regular inspection) as the expectation and understanding of the owner may differ from that of the veterinary practitioner.*” (VCI Newsletter, Issue 4, 2013). However, the standard of 24 h emergency care that is provided can vary between veterinary practices, and it is not always clear to the public what services are available, particularly in remote areas [6].

Within the academic literature, there is a dearth of empirical research on referrals and 24 h emergency care and most are based on expert opinion. Block and Gloss outline recommendations from a US veterinary committee process on responsible referrals and second opinions [7], some of which are summarised in Table 1. Moreover, little attention has been paid to the ethics of referrals and second opinions. American philosopher Bernard Rollin is of the opinion that the general veterinary practitioner “*has a moral duty to refer and defer to greater expertise*” ([8], p.717) and David Main advises that a veterinarian is expected to suggest referral to another practice if that would offer the chance of a better treatment, even at the loss of income [1].

**Table 1:** Expectations and responsibilities of first opinion (referring) and specialist (referral) veterinarians

First Opinion (referring) Vet	Specialist (referral) Vet
A client should never be discouraged or prevented from obtaining a second opinion or referral.	Specialists have a responsibility to determine whether particular patients should or should not be referred for a second opinion.
Patients’ records should be written legibly or typed and medical errors should be documented.	The specialist has a responsibility to communicate the status of patient to the referring vet.
Clients have an ethical and/or legal right to their animals’ medical records, and these should be voluntarily provided in a timely fashion on request.	Specialty practices should consider calling the day before to request records on any patient that is being referred and for which there is not yet referral information
Every effort should be made to provide this information so that it is readily available at the time of the initial referral.	Specialists have a responsibility to educate referring veterinarians in cases when they believe animals may or should have been managed differently.
First opinion vets should respect the time of the specialist they consult and not attempt to manage a case by telephone when referral would be a wiser course.	Specialists have a responsibility to consider referral to another hospital if they are unable to obtain a definitive diagnosis or effect successful treatment.

Adapted from Block and Ross [7]

Within a wider research project, this is the first in a series of three case studies exploring ethical challenges faced by veterinary professionals in Ireland. Building upon the results from a Policy Delphi study [5], here we aim to provide a detailed account of the constraints and possible opportunities to two prominent veterinary clinical services in Ireland: referrals/second opinions and 24 h emergency care.

## Methods

### Focus Groups

At a research workshop exploring veterinary ethical challenges in Ireland, held on 18 June 2015, eight stakeholders agreed to participate in two consecutive focus group sessions, one on referrals and second opinions (2 h duration) and one on 24 h emergency care (1.5 h duration). Purposive sampling of participants was used to reflect the diversity of veterinary clinical services available in Ireland. Selection criteria included seniority, experience with veterinary clinical services and an active role with a relevant veterinary organisation. Stakeholders included four referral veterinarians (from equine, farm animals and small animals), a referring mixed practice veterinary practitioner, a representative from the regulatory body, a member of the public and a senior member of an animal charity (Table 2).

**Table 2:** Participants in focus groups regarding veterinary clinical services (VCS)

	Gender	Stakeholder
VCS-1	F	Small Animal Practice-Referral Vet
VCS-2	F	Equine Practice-Referral Vet
VCS-3	M	Small Animal Practice-Referral Vet
VCS-4	M	Mixed Animal Practice-Referring Vet
VCS-5	F	Veterinary Regulatory Body
VCS-6	M	Farm Animal Practice-Referral Vet
VCS-7	F	Member of the Public
VCS-8	F	Member of Animal Charity

The sessions were moderated by the first author (MMS), and audio-recorded for qualitative analysis. An interview guide had been developed by the first author (MMS), discussed with co-authors, and revised until final agreement was reached. A semi-structured approach was used to guide the conversation towards the research questions. In the morning session, each participant was asked to list the three main challenges associated with veterinary referrals and second opinions, and to share their views with the group. This was followed by a collective exercise of appraising one of the first accounts on veterinary referrals [9] and to explore the differences with modern referrals. The afternoon session started with a group discussion around a vignette, validated elsewhere [10], describing a case scenario involving 24 h emergency care (Table 3), followed by an investigation of the barriers and the necessary strategies to improve the level of 24 h care. After the event, a summary with the main conclusions was sent to participants for comment and clarification.

**Table 3:** Vignette, used in focus group session, describing a case scenario on 24-h emergency care

Emma runs a small animal clinic in Co. Dublin. Podge, a cat with mega colon has been admitted for surgery. The owner is upset about leaving Podge and Emma reassures her, explaining that all pets are provided with ‘overnight care’ (e.g., automatic infusion pump, water or food). Emma omits to say, however, that animals are generally left unattended during the night, from 10 pm (time of the last medication) until 8 am.	

### Data handling and analysis

The sessions were transcribed verbatim, anonymised and inserted into NVivo 10, a qualitative research analysis software (© QSR International 2013). Thematic analysis was conducted using the data immersion/reduction technique [11]. As an initial deductive step, the research questions were used to sort and categorise the data according to two thematic, predetermined areas (i.e. constraints and opportunities). An inductive approach was then applied and a preliminary list of themes was generated after the initial coding, run by MMS and discussed with the senior author (AJH). The list of themes was refined in the following coding runs, while adding subthemes. The process was repeated iteratively until a final agreement was reached.

## Results

Six overarching, interrelated themes emerged which reflect the drivers and constraints involved in veterinary referrals/second opinions and 24 h care/cover. In the following sections, we consider these themes in some depth.

### Current guidance

The need of additional (and improved) guidance regarding referrals and 24 h care was highlighted. This theme was raised in discussions around the VCI-CPC which, at some instances, “*can be quite difficult to navigate and read*” (VCS-5). In this regard, it was mentioned that, in the VCI-CPC, the terms ‘referrals’ and ‘second opinions’ and the terms ‘24 h care’ and ‘24 h cover’ are often used interchangeably and that the difference between these terms is not always clear. With regard to the Veterinary Council of Ireland Premises Accreditation Scheme (VCI-PAS), concerns arose that current guidance might not provide sufficient clarity to ensure adequate provisions for 24 h emergency care. This view surfaced mainly in relation to the concept of hospitalisation, as illustrated in the following dialogue:

*The aspiration of the current Premises [Accreditation] Scheme is that it would be interpreted that if you are a hospital you will give night cover. Not defer night cover. That's the aspiration, but (…) as it is written in the regulations allow for the interpretation that the hospital can get its hospital status without [hospitalisation].* (VCS-4)

*That is wrong.* (VCS-8)

*But how do we use an inappropriate terminology? I mean, we know what people think what hospital means. It’s a commonly used word and we are using it inappropriately.* (VCS-1)

The suggestion was made to review the existing guidance and to ensure that veterinary hospitals have a veterinary professional (such as a registered nurse) providing 24 h cage-side care at all times.

### Clients’ expectations

A recurrent theme in the focus group was a sense that veterinarians need to more effectively manage clients’ concerns and expectations. Since the public has become more demanding and has higher expectations regarding the adequate level of care, participants highlighted the need for open disclosure about the level of care that veterinarians are able to provide. This will help prevent undesirable outcomes since nowadays a client is “*far more likely to get on Facebook and say that guy is never available when I want him*” (VCS-8). This could involve educating clients so they may have reasonable expectations as to the level of care that can be provided depending on the type of premises (i.e., practice, clinic or hospital). One interviewee reflected:

*Again it goes back to the practice premises [VCI-PAS]. You’ve got different levels [of care] but the clients are not aware that there is a clinic, there is a hospital. The client says: ‘I go to a vet’. But vet could be just the basics and the vet might not be offering very much more than to go out to your cow.* (VCS-8)

Meeting clients’ expectations can also include addressing the feelings of anxiety and distrust that the client may be experiencing in the process of transferring the care towards a referral centre:

*You are now leaving your animal with someone you don’t know, someone you have no relationship with. For the owner it’s the unknown. And that can be quite stressful.* (VCS-7)

Participants were of the opinion that no limits should be set as to when to refer or what should be considered an acceptable standard of 24 h emergency care since these are case specific. However, veterinarians should build trust by allowing clients to be part of the decision-making process. Presenting the available options will help clients to “*trust that the professional you are dealing with knows his own limits*” (VCS-7). This aspect was highlighted in the provision of 24 h care; although clients wouldn’t “*expect someone to be a major surgeon in the middle of the night*” (VCS-7), they would still expect “*that there will be someone there [in a hospital] looking after their animal*” (VCS-8).

### Veterinarian well-being

As regards the level of 24 h emergency care, meeting the regulatory requirements and clients’ expectations need to be measured against concerns with personal well-being. In terms of work-life balance, it was noted that “*is not tenable that someone is on-call 24 h a day 7 days a week*” (VCS-1) and that under “*EU work laws you should probably have a certain number of hours of rest, and try to apply that*” (VCS-3). This concern is especially relevant in farm animal practice where “*it’s a tradition for so long [that] it is actually expected that [vets] do their nights and work the next day*” (VCS-6). In addition, the nature of farm animal practice makes it more challenging to charge a premium fee for out-of-hours service since “*there wouldn’t be the hospital system that you would have in small animals or equine*” (VCS-6). In the words of a mixed practice veterinarian, “*a calving is a calving is a calving. It lands at 4 am or it lands at 4 pm. You can’t go [charging the farmer extra fees]*” (VCS-4). The suggestion was made that veterinary practices should group together in order to share out-of-hours emergency duties. It was noted, however, that in areas with low density of available veterinary services, the grouping of practices can be more challenging or even unachievable.

### Financial issues

Participants alluded to the difficult task of managing clients’ expectations “*in terms of what the client perceives they want for their animal as against what they want to pay*” (VCS-3). If, on the one hand, referring veterinarians must ascertain that the client can afford the costs of referral (VCS-4), on the other hand, clients may not be as concerned about money as veterinarians might consider (VCS-1). In this regard, veterinarians can make assumptions regarding what the client is willing to spend which might deter them from offering a referral. One referral vet was of the opinion that:

*Vets think that money is an issue but often, for the client, is not about the money (…) there is that conflict that there is a relationship you have and you believe something about the client and it may well be true but it might not be*. (VCS-1)

Nonetheless, one veterinarian noted that “*the profession is fantastic in what it is doing to the animals in this country currently for a way too low fare*” (VCS-4). Moreover, participants were in agreement that the veterinary profession, across the board, should be more proactive in charging fees which are closer to the overall high standards of veterinary clinical services in Ireland, since “*the client is expecting to pay but the veterinary profession is afraid to put the bill forward”* (VCS-3). The member of the public reinforced this perception stating from the client’s perspective that:

*(..) this is a hospital and [my dog] has been taken care all night and he has been looked after and he is coming back with a great big grin on his face. And I am paying for it. And that is fine. And that is how it should be. It’s not to say that people should be fleeced but is to say that there’s a value on care and that’s not wrong*. (VCS-7)

### Timeliness of referral

In addition to the financial considerations, one of the main issues regards the timeliness of the referral since it “*will have a knock down effect on everything else*” (VCS-7). Referral veterinarians suggested that complex cases are often not referred soon enough and that animal welfare may become a concern. In this regard, referrals in internal medicine were described as being more demanding than surgical referrals because:

*(…) in surgery it is very clear when you have to refer if an animal is fractured. But for [internal] medicine is not always that clear where the problem lies. And I think that is very hard for practitioners. Nine times out of ten [the animal] may respond to treatment.* (VCS-2)

The referring veterinarian further highlighted why referrals are not always timely by mentioning that “*because no one looks over my shoulder […], a challenge to me is to remember the referral as a good option*” (VCS-4). Other reasons that may discourage a first opinion veterinarian in making the timely decision to refer include the fear of losing the client and concerns with the disclosure of medical errors since “*when you are referring a case you are referring all your mistakes*” (VCS-8). The case of equine practitioners at [an Irish County] was used as an example where “*they would be very reluctant to seek referral because of business they are going to lose”* (VCS-4).

### Conflicts between veterinary practices

Several conflicting situations between veterinary practices arising from the provision of veterinary clinical services were described. This includes the inadequate sharing of relevant information from the part of referring veterinarians, which potentially impedes the successful outcome of a clinical case. Improved information sharing, including personal insights about the case, can help the referral veterinarian “*have an idea of what the [referring]* *vet is thinking about*” (VCS-1), especially in a time where referrals are becoming more informal, instead of based on a letter of referral:

*What actually happens now is we lift the phone, we make contact with the secretary of [the referral practice]. Could X do Y? ‘Oh, yes, yes, they could’ (..) There would be a brief conversation, but before this is usually just: ‘thank you for seeing this case’.* (VCS-4)

Improved communication between veterinary practitioners can also help managing possible competing opinions regarding the referral follow-up, and how aftercare is communicated to the owner. From a referral practitioner’s point of view, the responsibility to communicate with the client lies on the referring veterinarian since “*the owner is not my client, the vet is my client*” (VCS-2). Another prominent conflicting situation that emerged from the group discussion involves the veterinary services provided by practices run by animal charities and how these practices relate with neighbouring veterinary practitioners. In fact, it was noted that animal charities “*do all the routine veterinary work - the neutering, the microchipping - and then they are not available to provide aftercare for the animal*” (VCS-1) which can end up with animals getting “*dumped into the veterinary practitioners*” (VCS-4).

## Discussion

By relying on a qualitative exploratory approach, this case study investigation aimed to provide a value-based reflection on two prominent challenges associated with veterinary clinical services in Ireland: referrals and 24 h emergency care. The findings were based on two focus group sessions with the same group of eight participants. Despite the small sample size, this is one of the first attempts to gather empirical evidence on these issues in the field of veterinary medicine since available evidence is mostly based on expert opinion.

In terms of guidance, the need for improvements in the VCI-CPC was identified, especially on how the terms should be better defined. On the same note, a recent investigation of European Codes of Professional Conduct emphasised how the VCI-CPC can often be a complex document, both in terms of formulation and overall structure [12]. Drawing from other jurisdictions, the RCVS Code of Professional Conduct states that “*a referral may be for a diagnosis, procedure and/or possible treatment, after which the case is returned to the referring veterinary surgeon, whereas a second opinion is only for the purpose of seeking the views of another veterinary surgeon*”.^1^ Although this guidance helps to clarify the distinction between ‘referral’ and ‘second opinion’, it does not resolve it. Additionally, other commonly used concepts such as ‘advice’ and ‘supersession’ also require a standard definition. In the case of 24 h emergency care/cover, different designations should be used to describe the duty of providing out-of-hours care in registered premises (24 h care) and the duty to be on-call and providing emergency first aid and pain relief (24 h cover).

An important finding from this study is that the present VCI-PAS may not ensure the provision of adequate 24 h care/cover services for all accredited premises. The PAS Registered Veterinary Hospital Standards states that “*continuous patient monitoring must be provided as necessary on a 24-h basis by a registered person*” (point 15.2) and that “*all hospitalised animals must be checked as necessary over a 24-h period*” (point 15.4). However, there is no agreement as to what ‘as necessary’ actually means and this study suggests that, at least in some accredited small animal hospitals, out-of-hours cover is being directed to other hospitals. This situation has the potential to generate conflict with clients, fails to protect animal health and welfare and jeopardises the reputation of the veterinary profession.

Measures are needed in order to increase the standard of 24 emergency care/cover and to bridge the gap between the level of care that can be provided and the expectations of the client. In the UK, and following a recent consultation process, the RCVS has revised its guidance on 24/7 emergency cover,^2^ partially because of the mismatch between what was expected by the public and the service that realistically could be provided [13]. In fact, UK veterinarians are now expected to provide clients with full information about their 24 h emergency service and to refer to another practice where appropriate aftercare can be provided. It seems reasonable to expect that a similar approach may be used in Ireland. Steps have already been taken by the VCI in this regard [Aideen Neylon, personal communication, 23 Sept 2015].

Participants highlighted that clients’ values and expectations, such as cost, trust, confidence and level of care need to be considered at the time of providing referrals or emergency care, and that these expectations can be addressed by means of proper communication. However, conflicts may arise because it is not clear who has the primary duty to communicate to the client (the referring or the referral veterinarian), and the VCI-CPC provides little guidance in this regard [12].

Others have advocated for improved communication as to promote optimal referral care [7]. However, evidence suggests that veterinarians may be communicating ineffectively with their clients [14, 15]. In fact, lack of clients’ trust due to poor communication has been described as one of the most pervasive problems in veterinary practice [16]. Moreover, improved communication can help prevent errors in veterinary practice [17], and a UK disciplinary case on emergency out-of-hours care [18] illustrates the importance of appropriate communication, if, and when, conflicting interests arise. In order to meet their clients’ expectations, veterinarians need to learn how to effectively communicate the value (i.e., service, goods) they are delivering [19].

Although some veterinary schools across the globe have been giving increased attention to communication skills training [20–22], important gaps still remain [23]. More efforts should be made to provide Continuing Veterinary Education (CVE) in communication skills and ethical decision-making for veterinary practitioners.

Financial issues emerged as a liming factor at the time of referring a case or providing appropriate 24 h care. Above all, participants contend that clinical services are being undervalued. Our results indicate two possible explanations: a) veterinarians often make assumptions regarding how much clients are willing to pay, and b) the veterinary profession is reluctant to charge in accordance with the standard of care that is currently provided. Using a similar focus group approach, Coe and colleagues have detailed the financial aspects of veterinary care and their results mirror our own; in their assessment, veterinarians also indicated that their services are undervalued as “*a result of having trained clients over time to expect inexpensive services*” ([24] p.1514). Furthermore, David Main is of the opinion that the veterinary profession should not feel embarrassed in recommending expensive treatments if these accord with the best interest of the animal, and that ‘X-raying pockets’ (i.e., trying to predict how much the client will want to pay) may violate clients’ autonomy by preventing a full disclosure of the treatment options that are available [1].

Referring and referral veterinarians have different roles and responsibilities, which need to be acknowledged [7, 8]. Since these roles are complementary, veterinarians need to be aware of their own values as well as others’ values, which can be promoted through improved guidance and education. This is in line with the results from a Policy Delphi consultation process where veterinary professionals in Ireland considered that guidelines, conferences, and CVE training were the preferred measures to address the challenges with veterinary clinical services [5]. The VCI can contribute to raising awareness by promoting both transparent and full disclosure of records between the referring and the referral veterinarian, including possible medical errors. This requirement should be stated more clearly in the Code of Professional Conduct.

Surprisingly little empirical research on medical referrals can be found in the literature. A recent qualitative study from Australia highlights the challenges associated with the transition of care, and suggests that communication skills are required in order to improve patient outcomes [25]. Examining experiences of referrals in human medicine, however, is of limited use since the main ethical issues at stake seem to differ from veterinary referrals. The ethics of medical referrals often focus on the conflicting financial interests arising from the exchange of patients between general hospitals and private practitioners [26, 27], which does not seem to apply to veterinary referrals. In addition, since issues of animal health and welfare are less regulated than those of human health and wellbeing, there is more ground for expressing personal values in the field of veterinary medicine. In the words of Rollin, “*veterinary medicine, paradoxically, is more of a ‘people’ profession than human medicine, where the legal system backs the doctor even if he or she must work through the parent or guardian. The veterinarian, conversely, must keep the client happy to be allowed to continue to treat*” ([8], p.718).

The present study is part of a wider workshop where participants were divided into three groups, on the grounds of their expertise, and some limitations should be acknowledged. Although selection criteria included the role of participants with veterinary clinical services, no representative of the farming community was present. Nonetheless, the group was sufficiently diverse in order to minimise cohort effect. Further, it was the role of the moderator to ensure that every participant had a chance to meaningfully contribute to the debate. Finally, extrapolations should be made with caution since the small number of participants involved in this study may not represent the full range of views of every stakeholder involved with veterinary clinical services in Ireland.

## Conclusion

Five main recommendations emerged from this study to address current challenges with veterinary referrals/second opinions and 24 h care in the Republic of Ireland:

1. The terms used in current guidance (VCI Code of Professional conduct and Premises Accreditation Scheme) need to be clearly defined, in particular key terms such as ‘referrals’, ‘second opinion’, ‘supersession’, ‘emergency cover’, ‘24 h cover’ and ‘24 h care’.
2. The different levels of veterinary care that are being offered should be made clear to the client. This is essential to address clients’ expectations and reduce the reputational risk to veterinary professionals.
3. Transparent and full disclosure of patient records between the referring and the referral veterinarian should be promoted. It is also important to clarify who should be responsible to communicate with the client, and how.
4. Education in communication and ethical decision-making to veterinary students and practitioners (CVE training) should be promoted.
5. Resources should be developed and communicated to help clients understand the value of veterinary services, so that the fees reflect the quality and quantity of care and treatment. Training in business management for veterinarians is required, particularly regarding fee setting.

## Footnotes

1 Supporting guidance on referrals and second opinions. http://www.rcvs.org.uk/advice-and-guidance/code-of-professional-conduct-for-veterinary-surgeons/supporting-guidance/referrals-and-second-opinions/. Accessed 12 Dec 2016.

2 Supporting guidance on 24-h emergency first aid and pain relief. http://www.rcvs.org.uk/advice-and-guidance/code-of-professional-conduct-for-veterinary-surgeons/supporting-guidance/24-hour-emergency-first-aid-and-pain-relief/. Accessed 12 Dec 2016.
